# Age-Related Treatment Disparities among Medicaid Beneficiaries with Metastatic Colorectal Cancer

**DOI:** 10.4172/2167-7182.1000134

**Published:** 2013-10-07

**Authors:** Heidi D Klepin, Janet A Tooze, Eun-Young Song, Ann M Geiger, Kristie L Foley

**Affiliations:** 1 Wake Forest School of Medicine, Winston-Salem, North Carolina, USA; 2 National Cancer Institute, Rockville, North Carolina, USA; 3 Davidson College, Davidson, North Carolina, USA

**Keywords:** Colon cancer, Metastatic, Medicaid, Chemotherapy, Comorbidity

## Abstract

**Objective:**

To evaluate the impact of age on receipt of chemotherapy among low-income individuals with metastatic colorectal cancer.

**Data Sources/Study Setting:**

North Carolina Medicaid enrollees with metastatic colorectal cancer diagnosed from 1999 to 2002 with colorectal as their only cancer (N=326).

**Study Design:**

Retrospective analysis using linked data from the North Carolina Cancer Registry and Medicaid claims.

**Data Collection/Extraction Methods:**

Outcomes were chemotherapy use within one year of diagnosis and time to initiation of chemotherapy. Cox regression models were fit to evaluate the association between chemotherapy use and age, stratifying for comorbidity, and adjusting for patient, community, and health services characteristics.

**Principal Findings:**

Compared to 67.4% of patients aged <70 years, only 26.2% of patients ≥70 years received chemotherapy. After adjustment, younger patients with and without comorbidity were more likely to receive chemotherapy than older patients (hazard ratio (HR)=2.27, 95% confidence interval (CI) 1.41-3.66 and HR=6.33, 95% CI 2.87-13.96, respectively). Among those who received chemotherapy, the median time to receipt was 53 days, and did not differ significantly by age or comorbidity.

**Conclusion:**

In this low-income cohort, older age was consistently associated with non-receipt of chemotherapy but not longer time to initiation of chemotherapy regardless of comorbidity status.

## Introduction

Colorectal cancer is the third most commonly diagnosed malignancy among men and women in the United States and is a leading cause of cancer-related death [1]. The incidence continues to increase with the aging of the U.S. population with approximately 40% of cases diagnosed in individual's ≥ 75 years of age [2]. A significant proportion of patients have advanced disease at diagnosis. Clinical trials have shown that chemotherapy improves survival and quality of life for patients with metastatic disease [3-7]. It is unclear, however, if chemotherapy is consistently administered in the community, particularly among low-income and older adults.

Few studies have focused on treatment patterns for metastatic colorectal cancer. However, there have been multiple studies in the non-metastatic setting which have highlighted factors associated with receipt of curative treatment for colorectal cancer. These factors can be categorized into characteristics that describe the patient, the community within which the patient lives, and the health care system. For example, patient-specific factors such as increasing age, race/ethnicity, and comorbidity have been associated with less aggressive treatment of locally advanced disease and with decreased survival [8-11]. Socioeconomic factors such as income are also linked with lower rates of chemotherapy use in the non-metastatic (adjuvant) setting [12,13]. Finally, health system characteristics such as availability of specialized treatment centers and surgical volume have been associated with surgical outcomes and survival [14,15]. Some of these factors may also play a role in treatment disparity in the metastatic setting.

Older low-income adults represent a particularly vulnerable population. Medicaid-insured patients by definition represent a low-income population. Previous studies have shown that Medicaid insured patients are less likely to receive adjuvant chemotherapy compared to Medicare beneficiaries [16] and have lower survival rates [17]. Multiple studies have also shown that older adults are less likely to receive chemotherapy for adjuvant treatment of colorectal cancer [9-11] despite clinical evidence suggesting benefit in selected older adults [18] and consensus guidelines supporting its use [19]. Few studies, however, have evaluated the impact of age and socioeconomic status on palliative treatment for metastatic disease. Here, we describe our analysis of statewide data to evaluate the impact of age on receipt of chemotherapy in the context of patient, community, and health care setting characteristics among Medicaid patients. Documenting the administration of standard treatments for elderly and low-income populations is an important step in the evaluation of quality of care in this growing and under-studied demographic.

## Materials and Methods

### Design and setting

We conducted a cohort study among the Medicaid population in North Carolina. The cohort was assembled retrospectively, with existing data used to reconstruct prospective follow-up. The Wake Forest University Health Sciences and Davidson College Institutional Review Boards approved the research in accord with assurances fled with and approved by the U.S. Department of Health and Human Services; both Boards waived the need for informed consent. In addition, the study underwent review by the North Carolina Division of Medical Assistance and the North Carolina Central Cancer Registry.

### Study population

Individuals included in this study were diagnosed from 1999 to 2002 with only primary metastatic colorectal cancer, with metastatic disease defined as a Surveillance, Epidemiology and End Results summary stage equal to distant. Subjects with a prior diagnosis of localized colorectal cancer were not eligible. To ensure the availability of cancer registry and Medicaid claims data for the analysis, additional inclusion criteria were as follows:1) North Carolina residency at time of diagnosis; 2) received most of their cancer treatment at a North Carolina medical facility; 3) a valid social security number; 4) evidence of Medicaid coverage in the 180 days after their colorectal cancer diagnosis with at least one claim post diagnosis; 5) claims data for one year prior to diagnosis with no gap in Medicaid coverage greater than 90 days during that year; and 6) no additional cancer diagnoses before or after colorectal cancer (sequence 0). A total of 326 individuals met eligibility criteria and are included in this analysis.

### Data sources

Patient-specific demographic and clinical data were retrieved from the North Carolina Central Cancer Registry and Medicaid claims data, which were linked by social security number. The North Carolina Central Cancer Registry is mandated by state law to register all incident cancer cases and first courses of treatment, and follows the requirements of the North American Association of Central Cancer Registrars. North Carolina Medicaid is an almost entirely fee-for-service entity that covers all adults participating in cash assistance programs, those 65 and older at 100% below the federal poverty level, and the disabled. Medicare claims in North Carolina during the study period crossed over to Medicaid claims, ensuring dual-eligibility patients have complete claims data in the Medicaid files.

Community and health services data were derived from several sources. Community characteristics were assigned from Census using standard geocoding methods to link patients' address at diagnosis from cancer registry data to a block group and associated summary data. Health services characteristics were identified from the American Hospital Directory and University Health System Consortium, using a facility identification number for the facility reporting a case to the cancer registry.

### Measures

The primary outcome of interest in this analysis was receipt of any chemotherapy, regardless of other therapies, within one year of diagnosis. We considered chemotherapy to have been administered if, within 365 days after diagnosis, at least one of the following Healthcare Common Procedure Coding Systems procedure codes appeared in any claim: anything in the Jxxx (except J1440-1, J1950, J2405, J2430, J2505, J2820, J3487, J8565, J9031, J9175, J9202, J9212-9, J9225-6, J9230, J9395), 96408, 96410, 96412, 96414, 96520; G0345 to G0363; Q0083 to Q0085; RC331 to RC335; S9329; and W8222. ICD-9 codes 99.25, V58.1×, V66.2, and V67.2 also were used to indicate receipt of chemotherapy.

Potential factors associated with the outcome were grouped into patient, community, and health services characteristics. We obtained the patient level characteristics age, sex, and race from the cancer registry. Age was evaluated as a categorical variable using 70 years as a cut point. Race/ethnicity was categorized as white or nonwhite; there were too few patients in sub-groups to further categorize nonwhite. We defined non-cancer comorbidity using diagnostic ICD-9 codes from Medicaid claims in the year prior to cancer diagnosis to assign each individual a score on the D'Hoore adaptation of the Charlson Comorbidity Index(CCI), a well-established measure of mortality risk for comorbid conditions based on ICD-9 codes [20,21]. Due to the distribution of comorbidity scores in our sample, the CCI variable was categorized into a score of 0, 1-2 and >2.

We defined the community characteristics percent in poverty and urban/rural residence based on block group-level Census data, using zip code for 54 (13.9%) patients with invalid street addresses. Percent in poverty was categorized into tertiles based on the distribution among eligible patients. The results of an analysis restricted to patients with valid street addresses did not differ from the results presented below (data not shown).

The health services characteristic of total annual surgical volume was obtained from the American Hospital Directory then categorized into tertiles based on the distribution among eligible patients. Health centers that belong to the University Health System Consortium (https://www.uhc.edu/) were designated as academic medical centers for this analysis. We were unable to separately examine the impact of treatment at an NCI-designated Comprehensive Cancer Center because such centers in North Carolina all belong to the University Health System Consortium.

We also included receipt of local therapy (surgery or radiation) as a covariate. Local therapy was defined by either or both of a code for surgery or radiation in the cancer registry within 365 days after diagnosis, and at least one code in the 77xxx series of the Healthcare Common Procedure Coding Systems procedure codes appeared in any claim.

### Statistical analyses

All analyses were conducted with SAS statistical software, version 9.3 (SAS Institute Inc., Cary, NC). Descriptive statistics and chi-square analyses were calculated to compare baseline characteristics across treatment groups. We used survival methods to evaluate the receipt of chemotherapy over time. This methodology was chosen to account for the high censoring rate, primarily due to mortality, seen in this patient population. Use of survival methodology in comparison to logistic regression, no longer considers patients “eligible” for chemotherapy once deceased.

The Kaplan-Meier method was used to estimate the proportion of participants who received chemotherapy over time by age and comorbidity status, and the median time to chemotherapy among those who received chemotherapy. Cox proportional hazard regression was used to evaluate receipt of chemotherapy within one year post diagnosis. Patients were censored at death, disenrollment from Medicaid or one year of follow-up. Hazard ratios with 95% confidence intervals are presented for unadjusted and fully adjusted models. We present models stratified by comorbidity due to the relationship between age, comorbidity, and receipt of chemotherapy. The fully adjusted model included all the patient, community, and health services characteristics described above, due to *a priori* beliefs that all these factors could be associated with receipt of chemotherapy. In exploratory analyses, year of diagnosis and comorbidity score (comorbidity model only) were added to the adjusted models as covariates in order to investigate their association with chemotherapy use. Additional sensitivity analyses were performed restricting eligibility to patients who survived 90 days from diagnosis in an attempt to eliminate subjects who might have had poor performance status and/or were too ill to receive chemotherapy. The proportional hazards assumption was tested using the cumulative sums of martingale residuals. We considered analytic approaches that could account for the potential clustering of community and health services characteristics. Unfortunately, the sample size made such analyses infeasible. There were 241 distinct communities and 82 distinct hospitals in our data, a large enough number relative to the sample size to suggest that clustering would be unlikely to influence our results.

## Results

**Figure 1:**
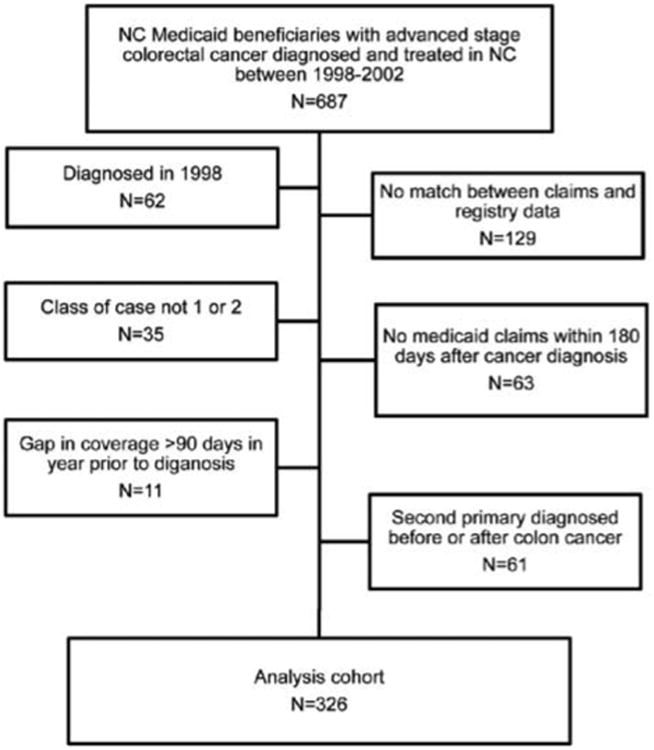
Study eligibility diagram. It depicts the eligibility criteria for the analysis cohort derived from North Carolina Medicaid beneficiaries diagnosed with colorectal cancer between 1999 and 2002.

Figure 1 illustrates the derivation of the analysis cohort. Among 625 Medicaid beneficiaries with advanced stage colorectal cancer, 326 met all eligibility criteria (52%). About 40% of the study population was over 70 years of age. Just over half (56.8%) of the study population was female and 49.4% were non-white. Less than half (35.9%) of the sample had non-cancer comorbidity at the time of diagnosis (CCI score ≥ 1). The minority (22.7%) of subjects were treated at an academic hospital. Approximately half (46.9%) of the cohort lived in a rural community.

Half (50.9%) of patients received chemotherapy with or without local therapy, while 32.2% received local therapy only and 16.9% received no treatment (Table 1). Compared to 67.4% of patients younger than 70 years, only 26.2% of patients ≥70 years of age received chemotherapy. A larger proportion of older adults received no treatment for their disease (23.8% versus 12.2%). Compared to younger adults, older adults were more likely to be female, have comorbidity (50.0% versus 26.5%), receive treatment at a non-academic institution, and receive treatment at lower volume surgical centers. The most common comorbid conditions differed by age (mild hepatic dysfunction [9%] and diabetes (10%) for patients <70 years versus congestive heart failure (21%) and diabetes (25%) for older adults).

Among patients with no significant comorbidity at colorectal cancer diagnosis, younger adults were more likely to receive chemotherapy than older adults (HR 2.27, 95% CI 1.41-3.66) after adjusting for patient, health care setting and community characteristics (Table 2). This finding persisted after restricting the cohort to only those patients surviving 90 days after diagnosis (HR 2.17, 95% CI 1.34-3.5). Patients who lived in urban versus rural communities were less likely to receive chemotherapy. Among patients with comorbidity, only age and poverty rank were associated with chemotherapy use in adjusted models. Specifically, younger patients were six times more likely to receive chemotherapy compared to older adults (HR 6.33, 95% CI 2.87-13.96). This relationship persisted in the model that was controlled for comorbidity score (HR 7.1, 95% CI 3.1-16.3), and in the model restricting the analysis to subjects who survived 90 days from diagnosis (HR 5.5, 95% CI 2.3-13.15). Patients in the highest poverty levels were three-fold more likely to receive chemotherapy (HR 3.02, 95% CI 1.25-7.30). We found no association between year of diagnosis and chemotherapy use on this cohort in exploratory analyses. Additional patient, health care setting, and community characteristics were not associated with chemotherapy use.

**Figure 2:**
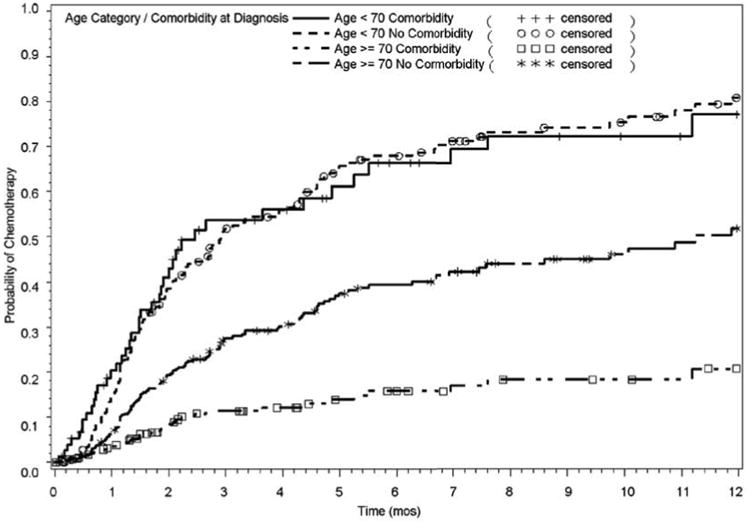
Probability of receiving chemotherapy within one year of diagnosis by age and comorbidity. It depicts the probability of receiving chemotherapy for metastatic colorectal cancer within one year of diagnosis by age and comorbidity among Medicaid recipients (N=326). Chemotherapy receipt is estimated for four groups: 1) younger adults (age<70 years) without comorbidity, 2) younger adults with comorbidity, 3) older adults (≥ 70 years) without comorbidity, and 4) older adults with comorbidity. The probability of receiving chemotherapy overtime is adjusted for demographics (gender, race), health care setting (treatment at an academic hospital, hospital surgical volume) and community characteristics (percent poverty and urban versus rural).

Cumulative incidence of chemotherapy also differed by age and comorbidity status. Specifically, the cumulative incidence rates of receipt of chemotherapy in 90 days were 52.3% for younger adults (<70 years) with no comorbidity and 48.6% for younger adults with comorbidity; in contrast, the 90-day cumulative incidence rates were 30.8% for older adults (≥ 70 years) without comorbidity and 19.9% for older adults with comorbidity. Among older adults, the cumulative incidence of chemotherapy within one year was 47.6% with no comorbidity and 28.5% with comorbidity; in contrast, the rates were 81.5% of younger adults with no comorbidity and 78.0% of younger adults with comorbidity. Results were similar for adjusted models (Figure 2). Among those who received chemotherapy, the median time to receipt was 53 days. We did not observe any significant differences in the median time when stratifying by age and comorbidity (log rank test p=0.62).

## Discussion

In this low-income cohort of patients with metastatic colorectal cancer, older age was associated with non-receipt of chemotherapy among patients with and without significant comorbidity. The presence of comorbidity influenced receipt of chemotherapy primarily among older patients. However, older age was not associated with longer time to receipt of chemotherapy from diagnosis among those who received chemotherapy. These findings suggest a significant age-related treatment disparity in receipt of chemotherapy.

The strong association between increasing age and decreasing use of chemotherapy has been documented in the treatment of non-metastatic colorectal cancer [9-11]. Schrag et al. [11] reported on adjuvant chemotherapy use for over 6,000 Medicare beneficiaries with stage 3 colorectal cancer and found that increasing age was associated with declining use of chemotherapy, independent of comorbidity and other factors [11]. For example, 78% of patients 65-69 years of age received adjuvant chemotherapy compared to 58% of those aged 75-79 years. Etzioni et al. [9] performed a systematic review of studies, which reported on adjuvant chemotherapy use for stage 3 colorectal cancer in the community setting [9]. Rates of chemotherapy use overall were 39-71% with increased age and comorbidity again strongly and independently associated with decreased treatment.

The impact of increasing age on chemotherapy use in the metastatic setting has not been well studied. An analysis of the National Cancer Database between 2000 and 2008 found that 21% of patients who presented with metastatic solid tumors received no anti-cancer therapy [22]. Across cancer types, both older age and low income were factors associated with lack of treatment. Specific to colorectal cancer, Hardiman and colleagues evaluated treatment of colorectal cancer and age using the Oregon State Cancer Registry [23]. For patients with metastatic disease, the vast majority under age 80 years received chemotherapy compared to approximately 10% of those over 80. These findings are consistent with our results. Similarly, only 29.3% of patients ≥ 75 years (versus 77.1% age<75 years) with advanced colorectal cancer received chemotherapy in a population-based study reported by the French Digestive Cancer Registry [24].

There are multiple potential explanations for decreased chemotherapy use with increasing age. Our finding that the presence of significant comorbidity alone does not account for age-related treatment disparity has been previously shown in the adjuvant setting [11]. Among advanced stage patients, Quipourt et al. [24] reported a significant age-related disparity in receipt of chemotherapy among a subset of patients with low comorbidity (CCI Score=0) in a French population-based cohort [24]. These findings are consistent with our results.

Our study goes further to show that the presence of comorbidity has a stronger association with treatments delivered among older adults compared to younger patients. We did detect differences in the most prevalent comorbid conditions among older versus younger patients in our cohort. Whether the type of comorbid condition was a greater determinant of treatment decision-making than age or number of comorbid conditions cannot be adequately assessed in this analysis. A retrospective study from the Netherlands found that the presence of comorbidity was a primary reason for withholding guideline adherent therapy for older adults with colorectal cancer and demonstrated significant differences in treatments offered to older versus younger patients [25]. The appropriateness of this practice pattern warrants further investigation, evidence suggests that higher comorbidity burden alone may not preclude chemotherapy benefits in the high risk adjuvant setting [26].

Some of the decreased chemotherapy use seen among older adults in our cohort may reflect unmeasured functional status impairments that may impact the tolerability of treatment. Functional impairments in older cancer patients can be present independent of comorbidity [27] or may be more pronounced in the presence of similar comorbidity among older versus younger patients. Impaired functional status, but not presence of comorbidity, has been previously shown to be associated with worse overall survival [28]. Such findings highlight the importance of accounting for functional status when assessing delivery of guideline-based therapy for older adults.

In addition to differences in comorbidity at diagnosis, the health care setting in which older adults were diagnosed and cared for differed from younger patients in this Medicaid cohort. Specifically, older adults were less likely to be treated at academic centers and more likely to be treated at institutions designated as lower volume surgical centers. These differences, however, did not explain the negative relationship between increasing age and decreasing chemotherapy use.

While our analysis accounted for multiple patients, community and health system level characteristics that could influence treatment patterns, multiple unmeasured factors may explain the relationship between age and treatment receipt. Patient preferences and limited health literacy may have influenced choice of management [29-31]. Older adults are equally willing to initiate chemotherapy [31], however, they may be more concerned about perceived treatment-related impairment in quality of life in the non-curative setting including risk of treatment-associated disability and cognitive decline [30,31]. Inadequate social support including absence of a care giver, and practical issues such as transportation can also impact treatment-decision making and [32] may represent a special challenge in a Medicaid population. Finally, age-related bias on the part of the health care provider may influence whether chemotherapy is offered to an older adult [30,33].

There are several limitations to this analysis. The potential for inaccurate coding exists for a claims-based analysis; clinical data from chart review were not available. Use of claims data may also underestimate comorbidity. We addressed this by our evaluation of claims data for comorbidity one year prior to cancer diagnosis. Another limitation to a claims-based analysis is the inability to capture the rationale for lack of treatment administration. This means that while we may raise the important issue of potential treatment disparity by age, we are unable to fully explain why this disparity might exist. In addition, claims data may not adequately capture use of oral chemotherapy such as capecitabine which was available in the latter aspect of our study. Use of state-specific data may be considered a limitation. However, state-specific analyses are preferred in Medicaid-based research given that Medicaid is partially state-funded and largely state-controlled. While differences exist across state programs, emerging literature in cancer disparities among Medicaid recipients in multiple states suggest that these studies have collective value and offer key insight into quality of care for low-income individuals. Similar to other retrospective cohorts, our analysis reflects treatment patterns from patients diagnosed several years ago. Despite this, we believe the implications of this research are still relevant. While there have been changes in the landscape of metastatic colorectal cancer therapy in the last decade, they have resulted in only incremental benefits in overall survival. 5-Flourouricil remains the backbone of therapy for metastatic colorectal cancer, and an acceptable standard option for older adults. Importantly, factors associated with initial treatment decisions among older adults and socioeconomically disadvantaged patients are underexplored.

This study also has several strengths. It is one of the few to evaluate treatment patterns for metastatic colorectal cancer, rather than locoregional disease. The use of claims data ensures a more complete description of treatments received than a cancer registry-based study, which might only capture planned treatment. Importantly, we evaluated treatment patterns for low-income but insured individuals by utilizing a Medicaid population who remain underrepresented in the literature. Thus, we can evaluate treatment patterns in a potentially disadvantaged population with insurance coverage. This study also allows comparison of patient characteristics in the context of the socioeconomic and health care setting, providing a unique glimpse at characteristics influencing treatment that would not typically be captured in clinical trials.

In conclusion, we believe the most important findings of our research are threefold: 1) older age was strongly and consistently associated with decreased use of chemotherapy in the metastatic setting; 2) additional patient, community and health care setting characteristics including comorbidity did not adequately account for age-related differences in treatment; and 3) the presence of comorbidity influenced receipt of chemotherapy primarily among older patients. Results from this study highlight the need to further investigate the impact of age on receipt of palliative therapies for colorectal cancer and to more carefully investigate the relationship between comorbid conditions and treatment outcomes in this setting.

## Figures and Tables

**Table 1:** Characteristics of Medicaid Beneficiaries with Metastatic Colorectal Cancer by Age (N=326).

	<70 years (N=196)	≥ 70 years (N=130)	
	N (%)	N (%)	p-value
**Treatment Received**			<0.001
Chemotherapy (+/- surgery/radiation)	132(67.4)	34(26.2)	
Surgery/radiation only	40 (20.4)	65(50.0)	
No treatment	24(12.2)	31(23.8)	
**Patient Characteristics**			
Gender			<0.001
Male	105(53.6)	36 (27.7)	
Female	91 (46.4)	94 (72.1)	
Race			0.79
White	98(50.0)	67(51.5)	
Black/Other	98 (50.0)	63(48.5)	
Charlson Comorbidity Index			<0.001
0	144(73.5)	65 (50.0)	
1-2	30 (15.3)	33(25.4)	
>2	22 (11.2)	32 (24.6)	
**Health Care Setting**			
Treated at an academic hospital			0.021
Yes	53(27.0)	21(16.2)	
No	143 (73.0)	109 (83.8)	
Surgery volume			0.002
Low (0-5500)	53(27.0)	59 (43.4)	
Middle (5700- 21000)	70 (35.7)	41 (31.5)	
High (23200-43900)	73 (37.2)	30(23.1)	
**Community Characteristics**			
% in poverty			0.86
Low (0-10.2%)	69(35.2)	42 (32.3)	
Middle (10.2- 19.2%)	63 (32.1)	43 (33.1)	
High (19.2- 68.6%)	64 (32.7)	45 (34.6)	
Urban/Rural			0.50
Rural	89(45.4)	64(49.2)	
Urban	107(54.6)	66 (50.8)	

**Table 2:** Characteristics associated with receipt of chemotherapy within one year of diagnosis among medicaid beneficiaries with metastatic colorectal cancer stratified by comorbidity burden (n=326).

	No Comorbidity (N=209)		Comorbidity (N=117)	
	Hazard Ratio (95% CI)		Hazard Ratio (95% CI)	
	Unadjusted	Adjusted*	Unadjusted	Adjusted*
**Patient Characteristics**				
<70 (versus ≥70 years old at diagnosis)	2.30 (1.46, 3.61)	2.27 (1.41, 3.66)	3.88 (1.93, 7.80)	6.33 (2.87, 13.96)
Male (versus female)	1.18 (0.83, 1.67)	0.97 (0.67, 1.41)	0.88 (0.46, 1.68)	0.56 (0.28, 1.13)
White (versus non-white)	0.95 (0.67, 1.35)	0.80 (0.54, 1.17)	0.66 (0.35, 1.24)	0.58 (0.29, 1.18)
Received surgery or radiation (versus no surgery or radiation)	1.01 (0.63, 1.63)	1.15 (0.69, 1.92)	1.71 (0.79, 3.72)	1.60 (0.70, 3.67)
**Health Care Setting**				
Treated at an academic hospital	0.66 (0.43, 1.03)	0.68 (0.40, 1.16)	0.65 (0.27, 1.55)	0.49 (0.18, 1.35)
Surgery volume				
High (versus low)	0.89 (0.57, 1.39)	1.08 (0.63, 1.86)	1.28 (0.54, 3.05)	0.79 (0.29, 2.15)
Middle (versus low)	1.36 (0.89, 2.07)	1.35 (0.87, 2.10)	2.13 (0.96, 4.71)	1.85 (0.76, 4.52)
**Community Characteristics**				
% in poverty				
High (19.2- 68.6%)	1.10 (0.71, 1.71)	1.06 (0.65, 1.72)	1.21 (0.56, 2.62)	3.02 (1.25, 7.30)
Middle (10.2- 19.2%)	1.61 (1.05, 2.48)	1.39 (0.89, 2.16)	0.99 (0.44, 2.22)	2.62 (1.00, 6.85)
Urban (versus rural)	0.62 (0.44, 0.88)	0.65 (0.43, 0.98)	1.14 (0.61, 2.13)	0.89 (0.45, 1.76)

* Adjusted model includes all variables presented in the table

## Abbreviations

CCI: Charlson Comorbidity Index
CI: Confidence Interval
HR: Hazard Ratio
ICD-9: International Classification of Diseases-9
NCI: National Cancer Institute
